# Toxic Epidermal Necrolysis-Like Acute Cutaneous Lupus Erythematosus Following an Upper Respiratory Infection

**DOI:** 10.7759/cureus.81798

**Published:** 2025-04-06

**Authors:** Matthew J Yan, Huy Phan, Sherwin Hsu, Yuna Kang, Yang Yu

**Affiliations:** 1 Dermatology, David Geffen School of Medicine, University of California Los Angeles (UCLA), Los Angeles, USA; 2 Internal Medicine, Olive View University of California Los Angeles Medical Center, Sylmar, USA; 3 Pathology, David Geffen School of Medicine, University of California Los Angeles (UCLA), Los Angeles, USA; 4 Dermatology, Olive View University of California Los Angeles Medical Center, Sylmar, USA

**Keywords:** cutaneous manifestations of systemic disease, stevens-johnson syndrome, systemic lupus erythematosus, toxic epidermal necrolysis-like acute cutaneous lupus erythematosus (ten-like acle), upper respiratory tract infections

## Abstract

Toxic epidermal necrolysis-like acute cutaneous lupus erythematosus (TEN-like ACLE) is a rare and severe form of cutaneous lupus erythematosus with an unclear etiology. We present a case of a 49-year-old woman with a past medical history of systemic lupus erythematosus, rheumatoid arthritis, and Sjögren’s overlap syndrome who developed TEN-like ACLE following a week-long upper respiratory infection (URI). While there are reported cases of TEN-like ACLE associated with excessive ultraviolet light exposure and abrupt medication discontinuation, this case uniquely highlights the novel association between URI and TEN-like ACLE, suggesting that URI may act as a previously unrecognized environmental trigger and precipitating agent for this rare condition.

## Introduction

Toxic epidermal necrolysis-like acute cutaneous lupus erythematosus (TEN-like ACLE) is a rare and severe form of cutaneous lupus erythematosus (CLE), affecting less than 1% of patients diagnosed with CLE [1].

In TEN-like ACLE, the clinical presentation resembles toxic epidermal necrolysis (TEN), a life-threatening skin condition characterized by widespread skin detachment and mucous membrane involvement [2]. Despite similar clinical features, TEN-like ACLE represents a distinct clinical entity from TEN. Most cases of TEN are drug-induced reactions, whereas TEN-like ACLE is a manifestation of CLE without a clear underlying etiology. Diagnosis of TEN-like ACLE involves a thorough clinical evaluation, medical history, laboratory analysis, and confirmation by skin biopsy with hematoxylin and eosin (H&E) staining and direct immunofluorescence (DIF).

This case report details a 49-year-old woman with systemic lupus erythematosus (SLE), rheumatoid arthritis (RA), and Sjögren’s overlap syndrome who developed TEN-like ACLE following an upper respiratory infection (URI). This novel association prompts an exploration of URI as a potential environmental trigger for TEN-like ACLE, a hypothesis not previously reported in the literature. This case highlights the clinical evaluation and management of TEN-like ACLE, investigates the complex interplay between URI and autoimmune exacerbations, and contributes to the broader understanding of TEN-like ACLE pathogenesis.

This article was previously presented as a poster at the 2023 CalDerm Fall Symposium Meeting in Carlsbad, CA, United States of America, as Poster #51, on September 22, 2023.

## Case presentation

A 49-year-old woman with a medical history of SLE, RA, and Sjögren’s overlap syndrome presented to the hospital with a rapidly progressing diffuse rash and oral mucosal sloughing lasting one week, raising concerns for TEN-like ACLE.

She was diagnosed with SLE in 2020 based on clinical features and serologic findings, including antinuclear antibodies 1:2560 (speckled pattern), positive anti-Smith (SM), anti-SM/ribonucleoprotein, anti-histone antibodies, and a positive direct Coombs test with polyclonal IgG. RA was diagnosed based on positive rheumatoid factor and inflammatory arthritis affecting the small joints. Sjögren’s syndrome was confirmed by positive SSA (Ro) antibodies, with symptoms of xerostomia and keratoconjunctivitis sicca.

Before her presentation, the patient was in her usual state of health until she developed URI symptoms, including a cough, fevers, chills, generalized weakness, and a runny nose. These symptoms lasted for three days and did not improve with the use of Tylenol. On day 4, she developed a nasal rash and edema, which progressed to ulcers on her lips with crusting. The rash continued to progress over most of her face, including the malar area, with associated eyelid swelling. On day 6, the rash spread to her chest, back, arms, and legs. The rash was mildly itchy, and the patient endorsed difficulty swallowing due to the oral rash, which led to decreased oral intake. She also noted dryness of her eyes and periorbital pain due to swelling, but reported no changes in vision.

She had been compliant with her rheumatologic medications, including prednisone (7.5 mg/day), methotrexate (15 mg/week), and hydroxychloroquine (300 mg/day). She denied any new long-term medications, supplements, detergents, or skin products. She lives at home with her husband and daughter, is not currently working, and primarily spends time indoors. She reports no recent sun exposure, changes to her daily routine, new pets at home, recent sick contacts, or travel, and does not engage in outdoor activities regularly. Due to the onset of a progressive rash with oral involvement, the patient presented to the hospital.

**Figure 1:**
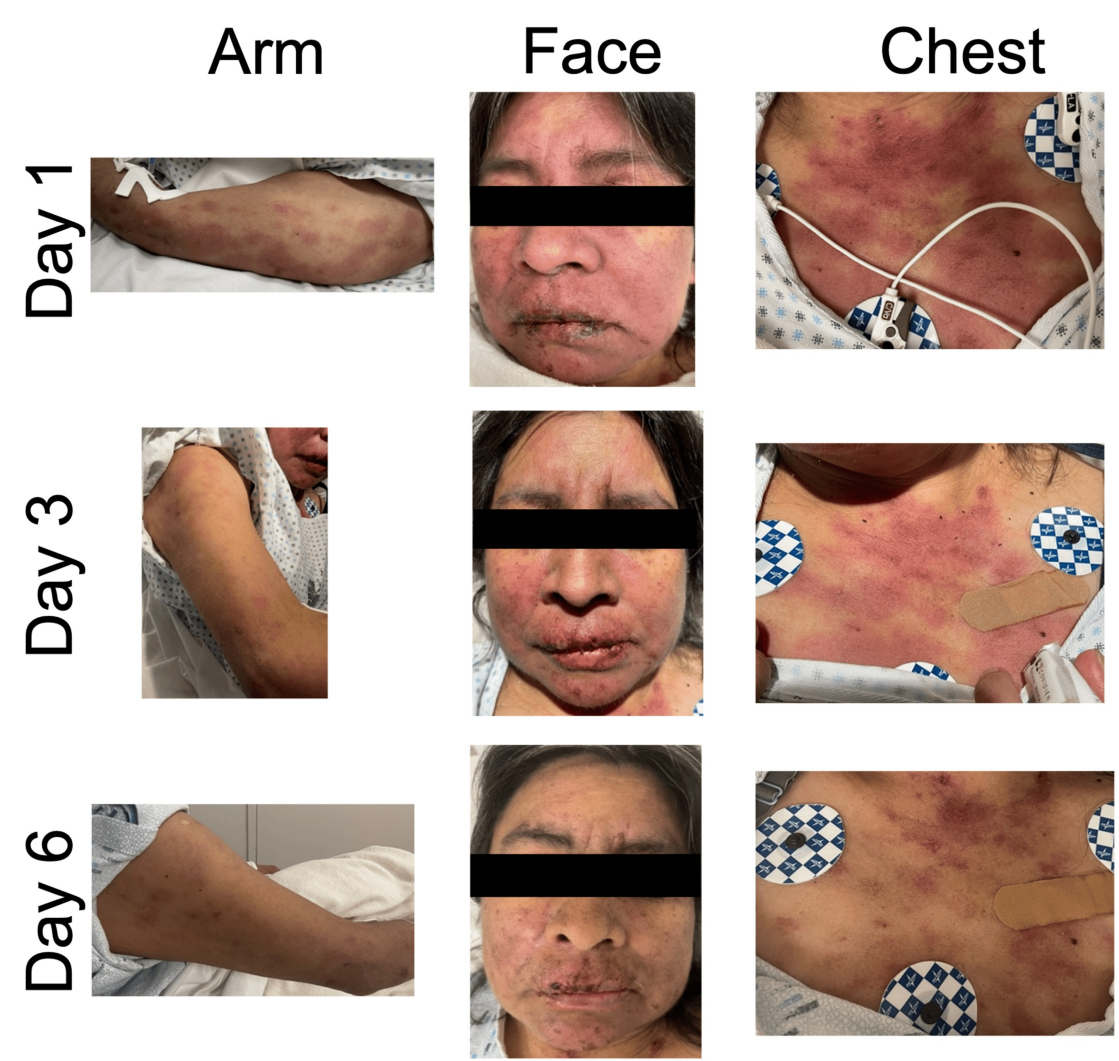
Dusky erythematous and violaceous patches and plaques on arms, face, and chest with oral erosions and ulcerations The progression of the rash is shown across days 1, 3, and 6 of the evaluation

On admission, the patient was afebrile with stable vital signs. Physical examination revealed a diffuse but photo-accentuated eruption of centrally dusky, erythematous, violaceous patches, and plaques on the chest, back, arms, legs, and face with oral erosions and hemorrhagic crusting, compromising 30% of the body surface area (Figure 1). Shearing of the epidermis was observed with lateral pressure applied to the skin on the chest, a positive Nikolsky sign. Examination of the upper respiratory system revealed fine, dry crackles on auscultation of the bilateral lung bases.

Given the patient’s past medical history of SLE overlap syndrome, laboratory tests were ordered. Results were significant for decreased C3 (49 mg/dL, normal: 90-180 mg/dL) and C4 levels (4.3 mg/dL, normal: 10-40 mg/dL) and elevated C-reactive protein (13 mg/L, normal: <10 mg/L) and erythrocyte sedimentation rate (73 mm/hour, normal: 0-20 mm/hour), consistent with an SLE flare.

**Figure 2:**
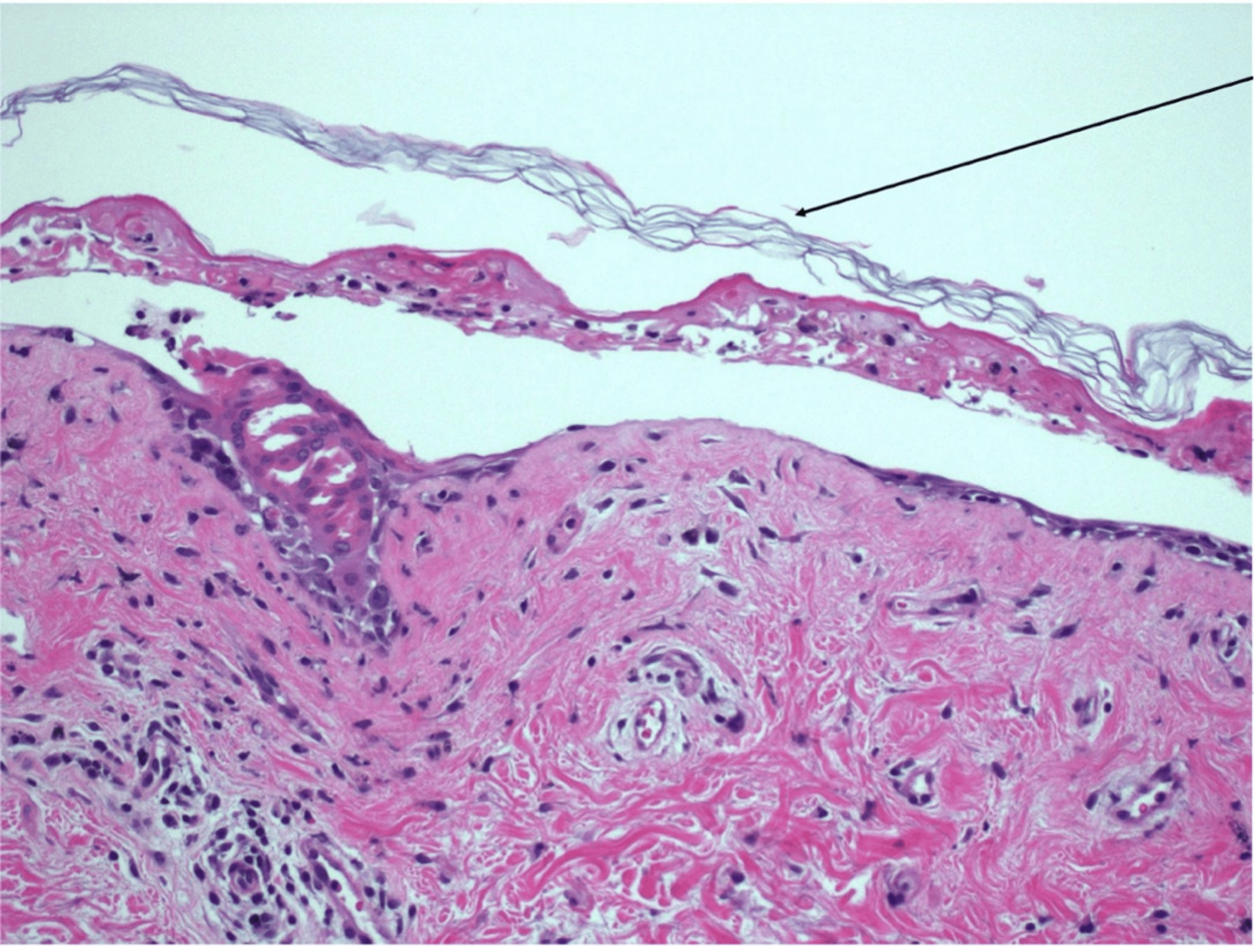
High-power view of a punch skin biopsy stained with hematoxylin and eosin (20×) Histology reveals full-thickness epidermal necrosis and detachment of the normal “basket weave” stratum corneum, as indicated by the black arrow. This pattern indicates rapid epidermal death and detachment

**Figure 3:**
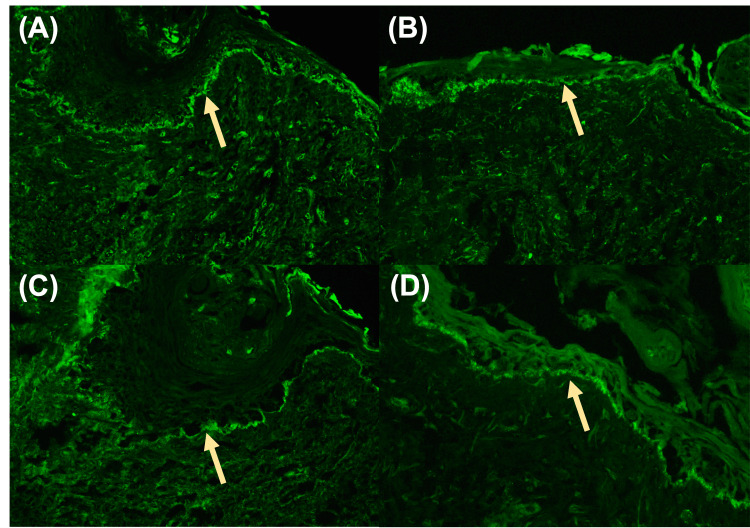
DIF staining of a punch biopsy from intact skin 1-2+ granular deposition along the basement membrane zone with antisera to IgG (A), IgM (B), IgA (C), and C3 (D). Yellow arrows point to the basement membrane zone with DIF positivity DIF: direct immunofluorescence; Ig: immunoglobulin

Two 4-mm punch biopsies were taken from the left chest for H&E and DIF analysis. H&E analysis revealed full-thickness epidermal necrosis and detachment of the normal “basket weave” stratum corneum consistent with Stevens-Johnson Syndrome (SJS) and/or TEN (Figure 2). DIF analysis confirmed lupus erythematosus with granular IgG, IgM, IgA, and C3 at the dermal-epidermal junction (Figure 3).

Blood cultures, respiratory cultures, and a respiratory viral panel testing for severe acute respiratory syndrome coronavirus 2, influenza A/B, and respiratory syncytial virus were collected on admission and returned negative.

The patient received a single dose of vancomycin (1,000 mg) and cefepime (200 mg) in the emergency department due to initial concerns for an infection. However, after her admission and reassessment, antibiotics were stopped, and she was treated with intravenous methylprednisolone (120 mg every 12 hours) and 30 g intravenous immunoglobulin (IVIG) (179 mg/kg/day) over three days and one dose of IV rituximab (1,000 mg) with significant improvement in her rash eruption and facial swelling over the course of five days (Figure 1). Given her improved clinical condition, the patient was eventually discharged on an oral prednisone course, starting at 40 mg/day for five to seven days, followed by a taper of 10 mg every week for four weeks until reaching her home dose of 7.5 mg/day.

## Discussion

SLE is a chronic autoimmune disease characterized by a malfunctioning immune system that mistakenly identifies healthy cells and tissues as foreign invaders, triggering inflammation and damage [1]. SLE commonly manifests in the skin, kidneys, heart, lungs, and brain, with symptoms of fatigue, malaise, fever, and skin rashes [3].

When SLE manifests in the skin, it is known as a CLE. In this case report, we describe TEN-like ACLE, a rare variant of CLE that carries an 11% mortality rate [1]. The clinical presentation of TEN-like ACLE resembles SJS/TEN. Both pathologies present with mucosal ulcerations in addition to widespread dusky erythematous areas of the skin with bullae formation and significant epidermal necrosis and detachment, which is often painful and pruritic [4,5].

Despite their similarities in presentation, TEN-like ACLE and SJS/TEN represent separate and distinct disease processes with clinical differences. SJS/TEN is usually categorized as a severe drug reaction because it is commonly triggered by medications (e.g., antibiotics and antileptics), inducing a type IV hypersensitivity reaction. Only rarely is a viral or bacterial infection implicated [1]. In contrast, TEN-like ACLE is a cutaneous SLE complication with a less-defined trigger and a poorly understood pathogenesis [6]. A combination of genetic, environmental (e.g., medication, infection, and ultraviolet, UV, light), and hormonal factors involved in the formation of immune complexes and subsequent complement activation is believed to play a role in aggravating lupus flares [6]. Furthermore, TEN-like ACLE is an autoimmune cutaneous manifestation of SLE, whereas TEN/SJS is commonly caused by an external vector.

Additionally, the timeline of symptom presentation generally varies and is another key distinguishing differential factor. TEN/SJS can present with a prodromal phase consisting of nonspecific, flu-like symptoms for one to three days that precede cutaneous symptoms [7]. Following the prodromal phase, TEN usually has an abrupt onset of widespread skin detachment-constituting a medical emergency requiring immediate attention. On the other hand, TEN-like ACLE typically has a slower and subacute progression of lesions over 14 days and is typically not considered an immediate medical emergency [1,7]. Thus, TEN-like ACLE is diagnosed through careful analysis of clinical features, histopathological examination of a skin biopsy, and exclusion of other potential causes.

Once TEN-like ACLE is diagnosed, the treatment goals are to modulate the overactive immune response and reduce inflammation. This is typically accomplished through administration of high-dose systemic glucocorticoids, such as prednisolone or methylprednisolone, and/or immunosuppressive agents such as azathioprine, mycophenolate mofetil, or methotrexate [8]. Additionally, IVIG, monoclonal antibodies, and supportive care have been shown to be effective in ameliorating flare-ups [8].

Our patient’s constellation of features, including mucosal involvement, Nikolsky sign, and H&E staining confirming full-thickness epidermal detachment of the skin, ultimately led to our differential diagnosis of TEN vs. TEN-like ACLE. The patient’s past medical history of SLE overlap syndrome, confirmatory DIF showing a lupus band (1-2+ granular deposition of IgG, IgM, IgA, and C3 along the basement membrane zone), reduced C3 and C4, and her predominantly photodistributed eruption collectively supported a diagnosis of TEN-like ACLE. While it is important to consider the possibility of a lupus band at this photoexposed location, which can persist even in the absence of active cutaneous lupus, the clinical presentation, including mucosal involvement, Nikolsky sign, and the pattern of epidermal detachment, overwhelmingly points to a diagnosis of TEN-like ACLE rather than a flare of cutaneous lupus [9].

Since our diagnosis of TEN-like ACLE was also found in the clinical context of the patient’s previous URI symptoms, special consideration was raised toward an associated URI-induced TEN-like ACLE, given her strict medication compliance and lack of other known triggers. Previous reports have described an infectious trigger precipitating SLE flares in other organ systems [4,10]. A review of existing literature suggests that starting a new medication can provoke TEN-like ACLE; however, in most cases, no information is provided regarding an etiology [1,11]. Thus, to our knowledge, there are no reported cases of a URI-induced TEN-like ACLE.

Possible mechanisms by which a URI could trigger TEN-like ACLE include molecular mimicry of self-antigens, epigenetic modifications of immune cells, and overproduction of proinflammatory cytokines [9]. URI was identified as the most likely trigger for our patient’s acute presentation, given that she also denied other possible precipitating factors such as UV radiation from sunlight or increased emotional stress levels, both of which have been previously shown to exacerbate underlying autoimmune activity [12].

## Conclusions

In this case report, we present a novel case of TEN-like ACLE following a URI. This case highlights the intricate pathophysiology of TEN-like ACLE, its severe clinical manifestations, and the frequent misdiagnosis with SJS/TEN. This case suggests that URI may serve as a potential environmental trigger for TEN-like ACLE, warranting further investigation into the mechanisms by which URI could contribute to the development of this rare condition. A better understanding of this potential link could lead to improved recognition and management of TEN-like ACLE in the future.
